# Superelasticity and force plateau of nickel-titanium springs: an *in vitro* study

**DOI:** 10.1590/2177-6709.21.3.046-055.oar

**Published:** 2016

**Authors:** Camila Ivini Viana Vieira, Sergei Godeiro Fernandes Rabelo Caldas, Lídia Parsekian Martins, Renato Parsekian Martins

**Affiliations:** 1 Private practice, Aracaju, Sergipe, Brazil.; 2 Assistant professor, Universidade Federal do Rio Grande do Norte (UFRN), Natal, Rio Grande do Norte, Brazil.; 3 Assistant professor, Director of Orthodontics program, Universidade Estadual Paulista Julio de Mesquita Filho (UNESP), School of Dentistry, Araraquara, São Paulo, Brazil.; 4 Adjunct professor, Universidade Estadual Paulista Julio de Mesquita Filho (UNESP), School of Dentistry, Department of Orthodontics, Araraquara, São Paulo, Brazil.

**Keywords:** NiTi, Superelasticity, Force plateau

## Abstract

**Objective:**

This paper analyzed whether nickel-titanium closed coil springs (NTCCS) have a different superelastic (SE) behavior according to activation and whether their force plateau corresponds to that informed by the manufacturer.

**Methods:**

A total of 160 springs were divided into 16 subgroups according to their features and activated proportionally to the length of the extensible part (NiTi) of the spring (Y). The force values measured were analyzed to determine SE rates and force plateaus, which were mathematically calculated. These plateaus were compared to those informed by the manufacturer. Analysis of variance was accomplished followed by Tukey post-hoc test to detect and analyze differences between groups.

**Results:**

All subgroups were SE at the activation of 400% of Y length, except for: subgroups 4B and 3A, which were SE at 300%; subgroups 4E and 4G, which were SE at 500%; and subgroup 3C, which was SE at 600%. Subgroup 3B did not show a SE behavior. Force plateaus depended on activation and, in some subgroups and some activations, were similar to the force informed.

**Conclusions:**

Most of the springs showed SE behavior at 400% of activation. Force plateaus are difficult to compare due to lack of information provided by manufacturers.

## resumo

**Objetivo:**

o presente artigo analisou se as molas helicoidais fechadas de níquel-titânio apresentam superelasticidade (SE), de acordo com a ativação, e se o platô de força medido corresponde ao informado pelo fabricante.

**Material e Métodos:**

160 molas foram divididas em 16 subgrupos, de acordo com suas características, e foram ativadas proporcionalmente ao comprimento da parte extensível (NiTi) da mola (Y). Os valores de força obtidos foram analisados para determinar as taxas de SE e os platôs de força, os quais foram calculados matematicamente - sendo esses platôs comparados aos informados pelos fabricantes. Uma análise de variância foi realizada, seguida do teste *post-hoc* de Tukey, para detectar e analisar as diferenças entre os grupos.

**Resultados:**

todos os subgrupos apresentaram SE em ativação de 400% do comprimento Y, com exceção dos subgrupos 4B e 3A (que apresentaram SE a 300%), dos subgrupos 4E e 4G (com SE a 500%) e do subgrupo 3C (que apresentou SE na ativação de 600%). O subgrupo 3B não apresentou comportamento superelástico. Os platôs de força dependeram da ativação e em alguns subgrupos, em determinadas ativações, foram semelhantes à força informada pelo fabricante.

**Conclusões:**

a maioria das molas apresentou comportamento superelástico na ativação de 400%. Os platôs de força são difíceis de ser comparados, devido à falta de informações por parte dos fabricantes.

## INTRODUCTION

In the 1980's, two new nickel-titanium alloys were suggested for orthodontic use, the "Chinese NiTi"^1^ and the "Japanese NiTi".^2^ These alloys had some advantages over the already existing nickel-titanium alloy used at the time, termed Nitinol;^3^ one was the fact that they did not obey Hooke's Law which determines proportionality between load and deflection of metals.

This is due to a transformation in the crystallographic structure from a martensitic to an austenitic phase, which can be induced by changes in temperature and/or stress.^4^ ^,^ ^5^ ^,^ ^6^ Since each of these two phases presents an inherent load-deflection rate, these alloys will behave differently depending on at which phase they are. Below a particular temperature termed "austenitic start" (A_s_), inherent to each alloy, the structure is completely martensitic; whereas above a second temperature termed "austenitic final" (A_f_) the structure is completely austenitic. At a given temperature above A_s_, a phase transformation could be induced by stress, transforming part of the alloy which is in austenitic phase into martensitic phase, e.g., when a nickel-titanium spring is activated,^2^ changing the alloy's properties.

When this transformation occurs, producing different load/deflection rates, and, when upon removal of stress, a reverse transformation takes place, producing a plateau in the curve of load/deflection, it is said that superelasticity (SE) occurred.

In Orthodontics, it is desired for a nickel-titanium closed coil spring (NTCCS) to be SE, in which the elastic modulus is low and the force is mostly constant. In this context, several manufacturers have produced NTCCS of different lengths and plateaus, but they need enough activation to produce sufficient stress to induce transformation or they will not be SE. Even though there is plenty of articles on NTCCSs,^7^ ^-^ ^11^ they lack information on how much activation is needed to induce SE.

Therefore, the aim of the present study is to determine the minimal activation for NTCCSs to be SE and to verify if the force plateaus measured are consistent with those informed by manufacturers.

## MATERIAL AND METHODS

Four groups of NTCCSs were created, according to the manufacturer, and further divided into 16 subgroups of ten NTCCS from the same batch, according to the length and plateau informed (Table 1).

**Table 1:**
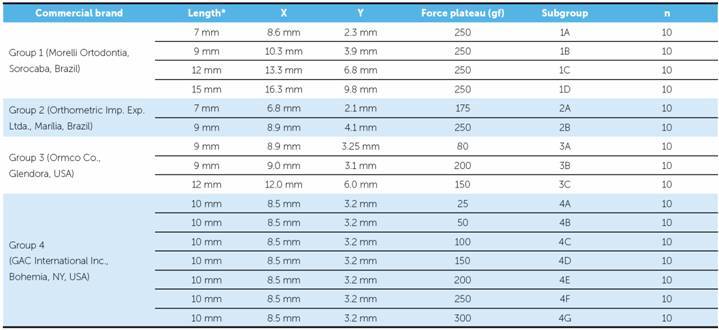
Commercial brands and groups division.

X = total length of the spring (from eyelet to eyelet); Y = length of the extensible part of the spring; * = length informed by the manufacturer.

**Figure 1:**
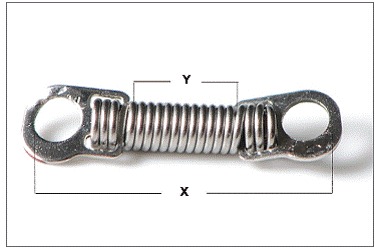
Nickel-titanium coil spring, the X dimension corresponds to the total length of the spring (from eyelet to eyelet, made os stainless steel) and the Y dimension is the length of the extensible part (NiTi) of the spring.

The size of each spring was measured with a digital caliper (Mitutoyo model SC-6, Suzano, São Paulo, Brazil), in order to obtain the total length of the spring (from eyelet to eyelet, made of stainless steel) (X), as well as the length of the extensible part (NiTi) of the spring (Y) (Fig 1).

Hooks adapted to a mechanical testing machine (EMIC DL2000, São José dos Pinhais, Brazil) were submerged in water at 37 ± 1°C (Fig 3),^12^ controlled with a heater (Termodelfim, São Paulo, Brazil) and a thermostat (Alife, São Paulo, Brazil). 

**Figure 2:**
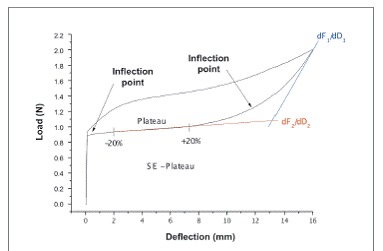
General load/deflection graph of a superelastic (SE) alloy. Indication of the inflection points and SE plateau (± 20%) and the first and second derivative (dF_1_/dD_1_ and dF_2_/dD_2_, respectively). Source: adapted from Segner et al,^13^ 1995.

**Figure 3:**
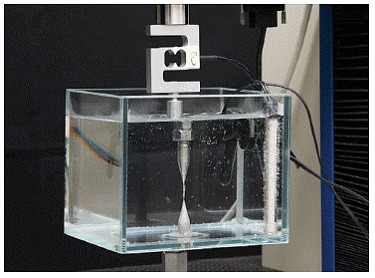
Glass aquarium attached to the testing machine.

Previously to each test, NTCCSs were adjusted at every 0.1 mm, so as to avoid any looseness that could compromise correct activation measurements.

The NTCCSs were activated to 100% of Y, deactivated and reactivated to increments of 100% until 1000% of Y. Tesc Software (EMIC, São José dos Pinhais, Brazil) registered deactivation forces at 20 mm/min in *.raw* format.

The plateau was the segment determined by two inflexion points calculated in the deactivation curve of the force data obtained (Fig 2). The value of the plateau was determined by the segment's midpoint^13^ and averages were compared to the information provided by the manufacturers, which were considered similar within a variation of 10%.

The first and second derivatives of force in relation to deformation were calculated in MATLAB R12(tm) software (The MathWorks Inc., Massachusetts, USA). The first derivative was located in the beginning of the deactivation curve, while the second one was located in the plateau - being determined in ± 20% of the plateau to minimize errors (Fig 2).^13^ These values were exported to Microcal Origin 8.0(tm) software (OriginLab, Northampton, USA) in which SE rates were calculated by dividing the first by the second derivative. NTCCSs were considered SE when the rate was above eight.

Since data were normally distributed, averages were calculated for SE rates and ANOVA with Tukey or Tamhane's T2 post hoc tests were used for comparison, performed with the aid of SPSS Software v.16.0 (SPSS Inc., Chicago, IL, USA). 

## RESULTS

**Figure 4:**
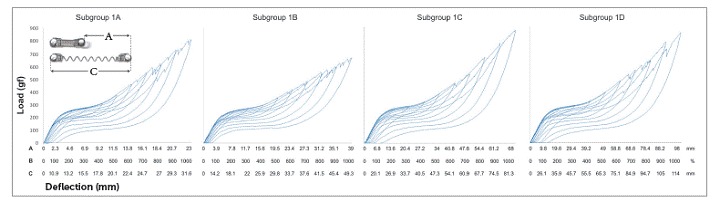
Load/deflection graph of one average spring of Group 1 (100 to 1000% of activation). On the axis of deflection, the amount of activation of the springs (mm) can be seen on the first line (A), the amount of activation corresponding to Y's percentage is on the second one (B), and the amount of activation of the springs added to its size is on the third line (C).

Only at 400% of Y activation (9.3 mm; 15.7 mm; 27.4 mm; 39.1 mm for subgroups 1A, 1B, 1C, and 1D, respectively) the NTCCSs of Group 1 were SE. They maintained that behavior up to 1000% of Y (Table 2 and Fig 4).

**Table 2:**
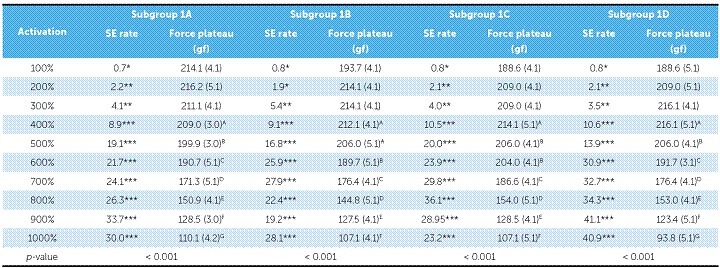
SE rate and force plateau values, mean (SD), of Group 1. Comparisons were only made for SE behavior.

*Non-SE behavior; ** Tendency to SE behavior; ***SE behavior. Similar groups show similar superscript letters

**Figure 5:**
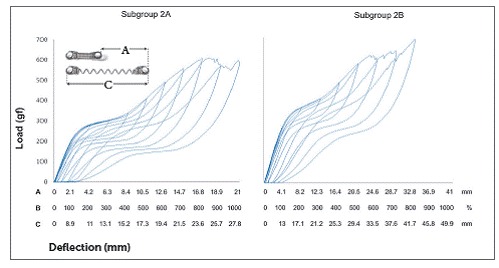
Load/deflection graph of one average spring of subgroup 2A (100 to 1000% of activation) and of subgroup 2B (100 to 900% of activation). On the axis of deflection, the amount of activation of the springs (mm) can be seen on the first line (A), the amount of activation corresponding to Y's percentage is on the second one (B), and the amount of activation of the springs added to its size is on the third line (C).

In Group 2, NTCCSs were SE at 400% of activation (8.4 mm and 16.6 mm in subgroups 2A and 2B, respectively) and above that activation (Table 3 and Fig 5).

**Table 3:**
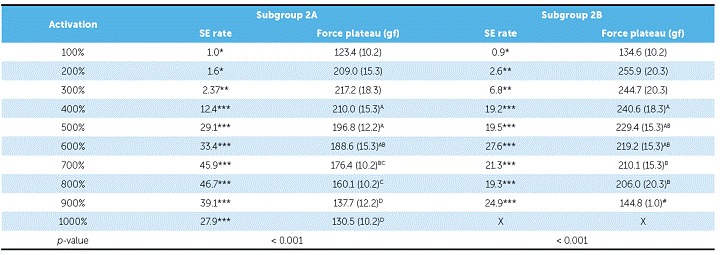
SE rate and force plateau values, mean (SD), of Group 2. Comparisons were only made for SE behavior.

*Non-SE behavior; ** Tendency to SE behavior; ***SE behavior. X = all springs ruptured, ^#^ not included on statistic due to n < 2.

Similar groups show similar superscript letters.

**Figure 6:**
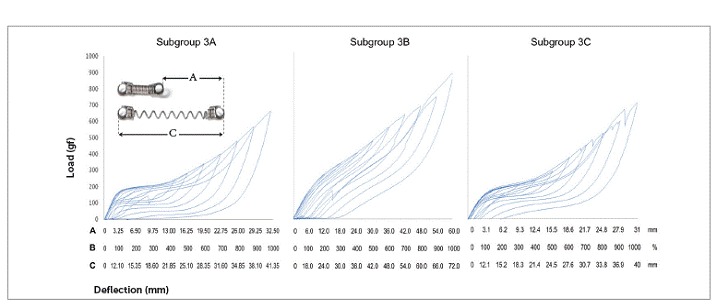
Load/deflection graph of one average spring of each of the subgroups of Group 3 (100 to 1000% of activation). On the axis of deflection, the amount of activation of the springs (mm) can be seen on the first line (A), the amount of activation corresponding to Y's percentage is on the second one (B), and the amount of activation of the springs added to its size is on the third line (C).

NTCCSs of Group 3 behaved very differently. While in subgroup 3A they were SE at 300% of activation (9.7 mm), in subgroup 3C, a minimum of 600% of activation (18.5 mm) was required. NTCCSs in subgroup 3B were not SE (Table 4 and Fig 6).

**Table 4:**
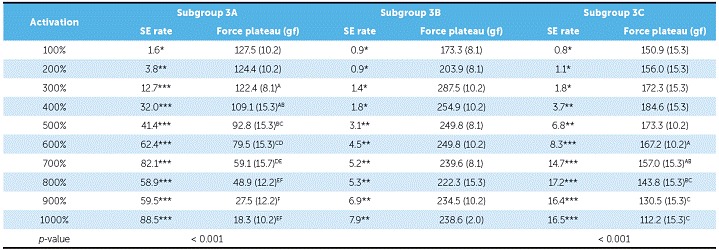
SE rate and force plateau values, mean (SD), of Group 3. Comparisons were made only among activations that induced superelasticity (according to the SE ratio) - subgroup 3B was not analyzed because NTCCSs did not show high enough SE rates.

*Non-SE behavior; ** Tendency to SE behavior; ***SE behavior. Similar groups show similar superscript letters.

**Figure 7:**
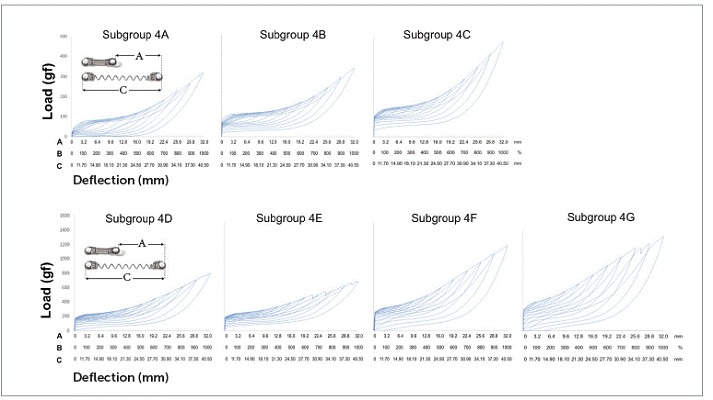
Load/deflection graph of one average spring of each of the subgroups of Group 4 (100 to 1000% of activation). On the axis of deflection, the amount of activation of the springs (mm) can be seen on the first line (A), the amount of activation corresponding to Y's percentage is on the second one (B), and the amount of activation of the springs added to its size is on the third line (C).

NTCCSs in subgroup 4C were SE at 300% of Y (9.6 mm) and above, while in subgroups 4A, 4B, 4D, and 4F SE started at 400% of Y (12.8 mm). Subgroups 4E and 4G were SE from 500% of activation on (16.0 mm) (Table 5 and Fig 7).

In general, SE rate increased with activation, but in subgroup 1A, it decreased at 1000%; in subgroup 1C it decreased at 900%, and in subgroup 1B, the rate decreased at 900% and increased at 1000%. In subgroup 2A, SE rate began to decrease at 900%, and in subgroup 2B at 700%. In subgroup 3B, SE rate did not decrease, while in subgroup 3C it began to decrease at 900%. In subgroup 3A, the rate showed a different behavior, in which it decreased from 800% and increased again at 1000%. In subgroups 4A, 4D, 4E, and 4G, the SE rate decreased at 1000%, while in subgroup 4C, it did not decrease. In subgroup 4B, the SE rate decreased at 900% and increased again at 1000%; while in subgroup 4F, the SE rate started to decrease at 700% and increased again at 800% and 1000% (Tables 2, ^3^, ^4^ and ^5^).

During 800% of activation, one NTCCS ruptured in subgroup 2A, two in subgroup 1D, and five in subgroup 2B. At 900% of activation, one NTCCS in subgroup 4G, two in subgroups 2A and 1D, four in subgroup 2B, five in subgroup 3A and eight in 3B ruptured. At 1000%, one NTCCS ruptured in subgroups 1D, 1B, 2B, and 3B, two in subgroups 3A and 4G, three in subgroup 2A, four springs in subgroup 3C, and five springs in subgroup 1C.

There were differences between all SE plateaus in Group 1 (Table 2). In subgroup 2A, the plateaus showed similar forces when activated at 400%, 500% and 600% of Y; at 600%, 700% and 800%, the plateaus were also similar, and at 900% and 1000%, the plateaus were different from themselves and from the other activations (Table 3). In subgroup 2B, the plateaus were the same from 400% to 600% of activation and from 500% to 800% of activation; and in subgroup 3A, the plateaus systematically decreased (subgroup 3B was not compared, since it was not SE) (Table 4). In Group 4, nearly all activations produced similar SE plateaus in lower activations and different SE plateaus in higher activations (Table 5).

**Table 5:**
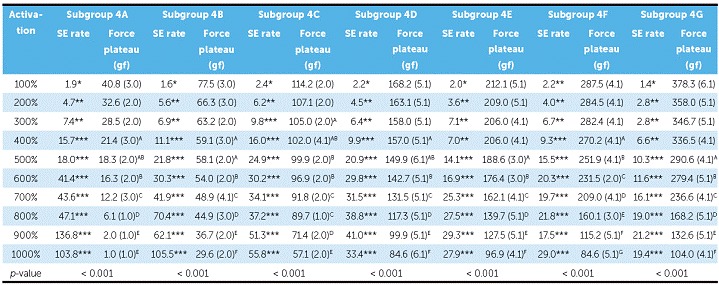
SE rate and force plateau values, mean (SD), of Group 4. Comparisons were only made for SE behavior.

*Non-SE behavior; ** Tendency to SE behavior; ***SE behavior; X = all springs ruptured. Similar groups show similar superscript letters.

The SE plateaus informed by the manufacturers (Table 1) of Group 1 and subgroup 3C did not correspond to the values observed in this study. For subgroups 3A, 4E, and 4A, NTCCSs produced a similar plateau to the informed one at 600%, 500%, and 400% of activation, respectively. In subgroups 2A, 2B, 4B, 4C, 4D, 4F, and 4G, the plateaus corresponded to the values provided by the manufacturer from 600% to 800%, 400% to 500%, from 600% to 800%, from 400% to 700%, 400% to 600%, from 400% to 600%, and from 500% to 700% of initial activation, respectively.

## DISCUSSION

In most subgroups (10/16), the springs were SE above 400% of activation. It should be reminded that the percentage of activation used does not regard the total length of the NTCCSs, as the majority of the papers published, but regards the length of the extensible part of the spring. Despite the knowledge that stress is a critical factor in using and measuring nickel-titanium, the literature does not describe how much NTCCSs should be activated in order to induce martensitic transformation by stress and, consequently, obtain a SE behavior.^11^ This information is critical, since low activation may not induce SE,^7^ ^-^ ^10^ ^,^ ^14^ ^,^ ^15^ ^,^ ^16^ which is in agreement with the present findings. Clinically, this is very important, since small springs, such as in subgroups 1A and 2A, will produce a plateau with smaller activations than what is normally used in Orthodontics (the average distance from canine to first molars is 23 mm),^17^ and even if a 1000% of activation equals to 23 mm, as in subgroup 1A, reverse transformation occurs only after some deactivation, only producing a plateau after 10 mm of deactivation (Fig 4). For that reason, NTCCS should be longer, but with non-SE materials, i.e., increasing the size of the eyelets. In medium length NTCCSs, such as subgroups 1B, 2B, 3A, 3C and all of Group 4, they should be overactivated as they are being engaged, returning to a smaller activation and securing them in place. On the same given example, a Group 4E NTCCS should be engaged, overactivated to 32 mm, returned to 23 mm and secured in place. Even then, some NTCCS would not produce a clear plateau and should be increased in size with non-SE materials, because 23 mm might fall a little short of the plateau, as in subgroup 3C at 1000% of activation. Long springs, such as subgroups 1C and 1D, should be hard to manipulate; in the same situation, they should be activated around 500-600% and upon return to 23 mm, engaged on the wire. Large activations would make the plateau end too soon, i.e., before the 7-mm space closure, while smaller activations would not produce SE. Since the NTCCS tested is very different, manufacturers should supply SE plateaus lengths, starts and finishes related to the amount of activation. 

**Figure 8:**
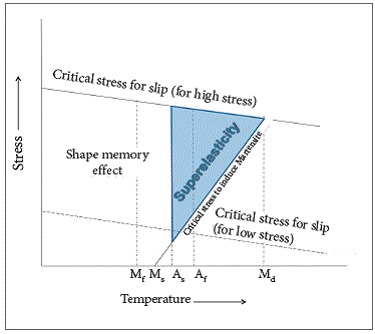
Graph of stress/temperature of SE alloys. M_f_ = martensitic final temperature, M_s_ = martensitic initial temperature, A_s_ = austenitic initial temperature, A_f_ = austenitic final temperature, M_d_ = temperature in which it is not possible for martensitic transformation to occur through stress

Source: adapted from Otsuka, Wayman, 1998.^20^

NTCCSs in subgroup 3B were not SE. This was possibly due to the fact that their alloy has transition temperatures higher than 37 °C. Since nickel-titanium martensitic transformation is a thermo/stress dependent phenomenon,^11^ ^,^ ^18^ ^-^ ^19^ whenever it cannot be induced, it is possible that either activation was insufficient to induce transformation (present results show SE rates increasing with activation), or that the temperature was lower than the required for a transformation to be stress-induced (M_s_) and also lower than the temperatures in which SE may be induced by transformation (A_s_)^20^ (Fig 8). Even with the American Dentistry Association (ADA) regulating that all SE material should be tested at 37 °C,^7^ ^,^ ^9^ ^,^ ^11^ NTCCS may not exhibit SE at this temperature. Therefore, manufacturers should be able to control transition temperatures properly. Even though an activation larger than 1000% of the NTCCSs of this subgroup (3B) could induce phase transformation, the possibility of rupture would be high (only one spring did not rupture at that activation).

Only 4% of the springs in Group 4 ruptured, compared to 80% of rupture in Group 2, 67% of rupture in Group 3, and 17% of rupture in Group 1. These findings, along with the activation required to produce phase transformation, limit the amount of activation of the NTCCSs. This has not been reported by the literature, even though rupture may not be the only factor limiting activation.

The minimum percentage values that create enough stress to cause stress-induced martensitic transformation can be calculated back into NTCCSs length and round to the next millimeter, if clinicians want to use those values clinically in order to use the springs' SE properties. It should be reminded that those values are the least amount that NTCCSs need in order to be activated, and that they should also be overactivated in order to avoid the fast drop of force that occurs upon the start of the reverse transformation from martensite back to austenite just before the plateau starts (Fig 2). Subgroup 1A NTCCSs (7 mm in length) should be activated at least 9.2 mm; subgroup 1B (9 mm), 15.6 mm; subgroup 1C (12 mm), 27.2 mm; and subgroup 1D (15 mm), 39.2 mm. Subgroup 2A (7 mm) should be activated at least 8.4 mm and subgroup 2B (9 mm) at least 16.4 mm. Subgroup 3A (9 mm) should be activated at least 9.75 mm and subgroup 3B (12 mm), 36 mm. Finally, Group 4 (10 mm) should be activated at least 12.8 mm on subgroups 4A, 4B, 4D and 4F, 9.6 mm on subgroup 4C and 16 mm on subgroups 4E and 4G. It should be noted that longer NTCCSs will need activations that will be too large for the orthodontic parameters and will not have superelastic behavior in the oral cavity.

SE rate increased with activation in all subgroups, but above certain activations in few subgroups, it either maintained itself or decreased (Table 3). When analyzing this table along with the load/deflection plots of average NTCCSs from each group (Figs 4, ^5^, ^6^ and ^7^), it can be observed that the lighter NTCCSs in Groups 3 and 4 showed higher rates with very smaller activations. That is probably due to thermal treatment which is commonly used to increase M_s_.^21^ ^,^ ^22^ This procedure will decrease the stress required to start martensitic transformation,^23^ thereby producing a lower SE plateau.^14^ ^,^ ^21^ ^,^ ^22^

SE plateaus varied with activation and, while these values did not correspond to the information supplied by manufacturer in some groups, in other groups it corresponded only at specific activations. This could be a problem when using nickel-titanium because, as demonstrated (Tables 2, ^3^, ^4^, and ^5^), plateaus change at different activations and the manufacturers do not provide the information of which activation will produce the plateau informed. The need for this information was suggested in the literature^8^ and is essential, so that orthodontists and researchers could select the most adequate NTCCS.

## CONCLUSIONS

1) Most subgroups showed a SE behavior above 400% of activation, except for subgroups 4B and 3A which exhibited it above 300%. Subgroups 4E and G, and subgroups 3C were SE above 500% and 600% of activation, respectively.

2) Subgroup 3B was not SE.

3) SE rates increased with activation, while force plateaus decreased.

4) Force plateaus provided by the manufacturers are difficult to compare, since initial activation alters it. Manufacturers should provide more specific information on their nickel-titanium CCSs.

5) NTCCSs are not always superelastic, especially at low activations. If SE is desired, NTCCSs must be activated at least 5 to 6 times the length of active part of the spring.
